# RNA Editing in Hepatitis Delta Virus: Unsolved Puzzles

**DOI:** 10.1100/tsw.2004.123

**Published:** 2004-08-13

**Authors:** Geetha C. Jayan

**Affiliations:** Division of Viral Products, CBER, FDA, Bethesda, MD 20892, USA

**Keywords:** RNA editing, post-transcriptional gene regulation, gene expression, adenosine deamination, ADAR, hepatitis delta virus

## Abstract

RNA editing, or post-transcriptional changes in the sequences of RNAs, is being increasingly recognized as an important player in the regulation of gene expression in vertebrates and invertebrates. Different types of RNA editing have been reported. This review discuss the type of RNA editing caused by cellular enzymes known as adenosine deaminases that act on RNAs (ADARs), and it's significance in the lifecycle of an RNA virus, hepatitis delta virus.

## INTRODUCTION

As implied by the term "editing", "RNA editing" refers to changes made on RNAs. Editing on RNAs was originally described by Benne and coworkers in 1986 as a unique phenomenon occurring on certain messenger RNA (mRNA) transcripts in the mitochondria of trypanosomes[1]. But subsequent studies have shown that this phenomenon occurs on mRNA transcripts of several species.

Now the term RNA editing is used to indicate certain programmed post-transcriptional processes, not including RNA splicing, that can alter the genomically encoded nucleic acid sequence of RNAs[2]. RNA editing and splicing differ from each other primarily in the fact that the former is a fine-tuning mechanism in which usually single nucleotide changes are effected, whereas in splicing, large pieces of nascent RNA are cut and pasted together[3,4].

When RNA editing occurs on mRNAs, it can cause reprogramming of genetically encoded messages and can alter the coding output of messages. The phenomenon of RNA editing, therefore, has added a new twist to the traditional tale of the two entities — the gene and the protein — in the gene expression pathway. Consequently, the conventional wisdom of deciphering amino acid sequences of proteins from corresponding gene sequences (the genetic code) has to be done with caution. It is not surprising, therefore, that RNA editing is now recognized as an important player in the gene expression pathway. Different forms of RNA editing that can be grouped under two major categories have been reported as shown in Fig. 1.

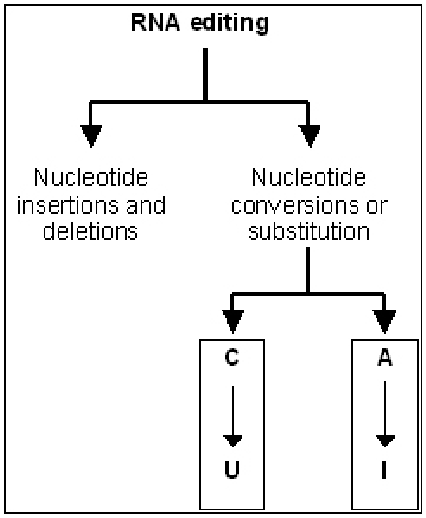

**FIGURE 1**. Classes of RNA editing. RNA editing can be grouped under two classes. (1) Nucleotide insertions and deletions: alterations on RNA sequences by the insertion of bases into existing RNA sequences or by the deletion of existing bases. (2) Nucleotide conversions or substitutions: alterations on RNA sequence by converting one base to another. Conversions of cytosine to uracil and adenosine to inosine are the known nucleotide conversions occurring in RNA editing. Adenosine to inosine conversion is caused by ADAR enzymes.

This review focuses on nucleotide conversions, specifically the conversion of adenosine (A) to inosine (I). These conversions involve a deamination reaction and are catalyzed by a group of enzymes known as ADARs (adenosine deaminases that act on RNAs)[5].

## THE ADAR ENZYMES

ADARs were originally identified from frog oocytes[6,7,8]. Subsequently, they have been isolated from several different species. In humans, three ADAR genes (ADAR1, ADAR2, and ADAR3) have been cloned and sequenced, and ADARs 1 and 2 are known to be catalytically active. All ADARs have a common domain structure (Fig. 2): N-terminal double-stranded RNA (dsRNA) binding domains (dsRBM) and a highly conserved C-terminal deaminase domain. The number of dsRBMs and the distance between these domains vary among the different types of ADARs[9,10]. Additionally, varied forms of each ADAR protein are also synthesized from multiple promoters[3,11], alternative splice sites[11,12,13], and alternative translation initiation sites[3,4]. The biological significance of these diverse versions of ADARs is not understood.

### Catalytic Activity of ADARs

The catalytic activity of ADAR enzymes resides in their C-terminal deaminase domain and their substrate specificity for binding to RNAs that have partial or complete double-stranded character comes from the N-terminal dsRBMs. The deaminase domains catalyze the hydrolytic C6-deamination of adenosines to inosines (Fig. 3). Technically, adenosines present on any dsRNA that is over 23 base pairs long can be deaminated by ADARs[14,15,16]. However, like other biological catalysts, ADAR does not act randomly on any substrate. Its activity on substrates is characterized by certain specific attributes: “preference” and “selectivity” are two important features/determinants of deamination by ADARs.

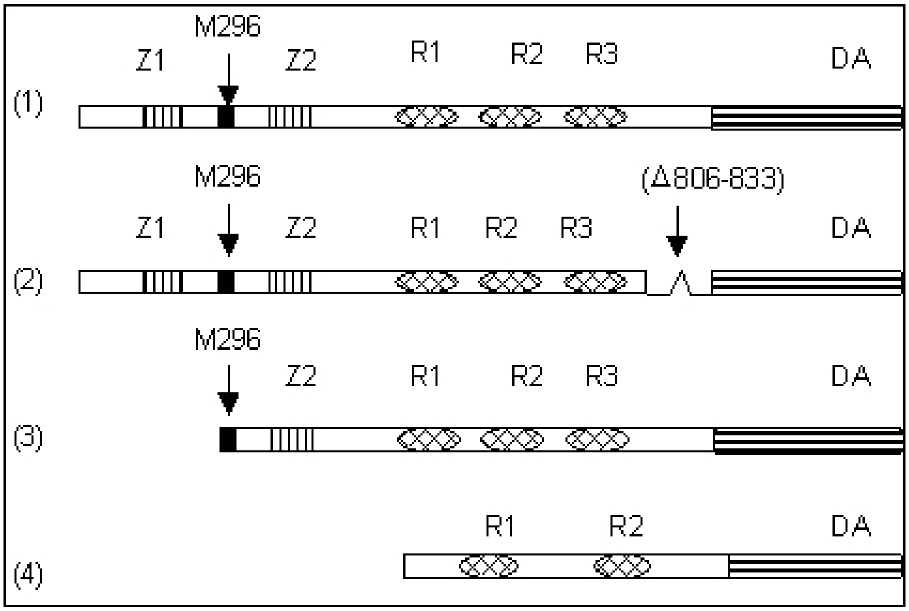

**FIGURE 2**. Structure of four of the different forms of ADARs. Z1, Z2: Z-DNA binding domains. R1, R2, R3: double-stranded RNA binding domains (dsRBDs). DA: deaminase domain. M296: Methionine at position 296. As shown here, all ADARs have N-terminal dsRBDs and a highly conserved C-terminal deaminase domain. The presence of Z-DNA binding domains, the length of the N-terminal region, the number of dsRBDs, and the amino acid sequences spanning the region between the dsRBDs and the deaminase domain vary among the different ADARs. (1) Full-length ADAR1a: transcription product of ADAR1 gene. It is a protein having 1226 amino acids, has 2 Z-DNA binding domains, and 3 dsRBDs. (2) Full-length ADAR 1b: transcription product of ADAR1 gene. It is similar to full-length ADAR1a, but has a deletion of 28 aminoacids (Δ 806 – 833) towards the C-terminus. (3) Truncated ADAR 1a (M296 – ADAR 1a): transcription product of ADAR1 gene. It is a shorter version of full-length ADAR 1a. Translation of this protein initiates from M296, and it has only one Z-DNA binding domain. (4) ADAR 2a: Transcription product of ADAR2 gene. It has no Z-DNA binding domain, and has only 2 dsRBDs.

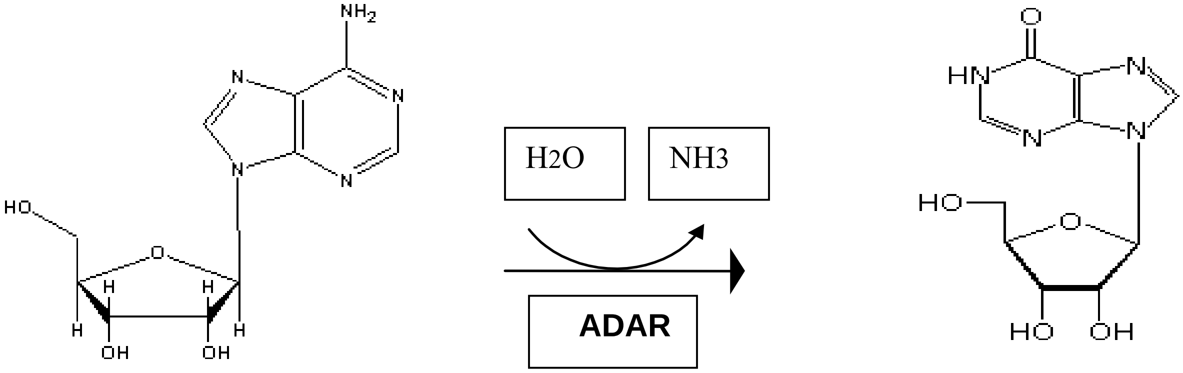

**Adenosine** **Inosine**

**FIGURE 3**. Catalytic activity of ADAR enzymes: ADARs catalyze the C6 deamination of adenosines on double-stranded RNA substrates to form inosines. The oxygen in water acts as a nucleophile, attacking the C6 of adenosine.

## *Preference and Selectivity of ADAR Enzymes*

On a dsRNA substrate, ADARs selectively target adenosines with an immediate 5′ neighbor of A, U, or C, but not G[17], and adenosines that are at least 8 bases upstream of the 3′ end of the RNA[17,18]. This selection of adenosine bases, depending on their location and neighboring sequences, is referred to as the "preference" of the enzyme[19]. "Preference" most likely arises from the physical characteristics of the ADAR enzymes and the ability of the enzymes' catalytic domain to access the C6-amino group on the adenosine[19].

Unlike "preference", "selectivity" of ADARs is a substrate-dependent feature: the secondary structure of substrate RNAs is the primary determinant. "Selectivity" indicates the extent to which a substrate can be deaminated. It is expressed as the percentage of adenosines in a dsRNA substrate that gets deaminated[19]. The higher the percentage of adenosines deaminated, the lower the selectivity, and vice versa. Based on "selectivity", ADAR substrates can be grouped under two categories:

- Substrates with low selectivity: these are completely base-paired dsRNAs in which 50% or more of the adenosines can be deaminated[20].
- Substrates with high selectivity: these are structured dsRNAs, such as those with mismatches, bulges, loops, and other nonbase-paired regions interrupting the double-strandedness. In these substrates, only up to 10–25% of the adenosines can be deaminated[17,21].

All the known biological substrates for ADARs have high selectivity, with deaminations targeted to specific adenosines.

## Biological Significance of ADAR-Catalyzed Deamination

ADARs convert adenosines on RNA molecules to inosines, and inosines are read as guanosines by the transcription and translation machineries[22]. Therefore, adenosine to inosine changes on mRNAs can alter the codon product of genes. These proteins products, which are translated from ADAR-edited messages and have altered amino acid sequences, may also differ from the native protein in their biochemical and biological properties.

Yet another consequence of adenosine to inosine substitutions on RNAs can arise from the property of inosine to base pair preferentially with cytosine and not uracil. Due to this base-pairing preference, adenosine to inosine changes can alter adenosine-uracil base pairs on structured RNAs to inosine-uracil mismatches, and subsequently alter the secondary structure of the RNA.

Therefore, adenosine to inosine RNA editing can potentially affect any biological process that is dependent on RNA structure and/or sequence.

### *Biological Substrates for ADAR-Catalyzed Deamination*

In mammals, only a few RNAs have been established as substrates for ADAR-catalyzed editing. These are all mRNA transcripts of the nervous system and require specific levels of selective ADAR-catalyzed deamination for physiological functioning of their protein products[4,23,24]. Typical examples are the mRNA transcripts for G-protein coupled serotonin receptors and for glutamate-gated ion channels. In these transcripts, selective ADAR-invoked editing activity at specific sites are essential to synthesize receptor proteins having normal neurotransmission properties[25,26,27].

It is quite intriguing that all the mammalian ADAR substrates reported thus far are brain-specific transcripts, even though ADAR expression has been reported in several tissue types[9]. Probably, benefits derived from the occurrence of editing events have most significance to the nervous system: the two major advantages of using RNA editing to diversify the coding capacity of genes over genetic imprinting of favorable mutations are the freedom to regulate the edited transcripts quantitatively and the freedom to enforce temporal and spatial control over the edited products[23]. From a biological standpoint; it is conceivable that such regulatory switches are more warranted in the nervous system than in other systems in our body.

However, more recent studies have reported inosine-containing polyA-mRNAs in several tissue types such as heart, muscle, thymus, and lungs[28]. Therefore, it is possible that nonbrain mammalian ADAR targets will soon find their way into the world of ADAR substrates.

In addition to mammalian ADAR substrates, certain double-stranded viral RNAs are also known to be substrates for ADARs. In this context, it is hypothesized that ADAR enzymes may be part of the cellular antiviral defense pathway, by facilitating the formation of inosine-containing RNAs that can be subsequently degraded by inosine-specific nucleases[29,30].

Contrary to this increasingly popular antiviral hypothesis about ADARs, certain viral RNAs have developed strategies to utilize the host ADAR enzymes to their advantage. A well-known, and in fact the only established, example for such a viral pathogen whose lifecycle is benefited by ADARs is hepatitis delta virus (HDV).

## HDV

HDV is a unique subviral human pathogen that requires hepatitis B virus (HBV) to complete its infectious lifecycle. It increases the risk of fulminant hepatitis in patients and carriers[31] of hepatitis B, and is responsible for the most severe form of viral hepatitis[32]. Therefore, HDV is a major health risk factor in HBV patients and in populations that have high carrier rates for HBV[31]. This risk is aggravated by the fact that there is no effective therapy against HDV infection or HDV pathogenesis[33,34].

## Taxonomy

HDV is the only member of the virus family Deltaviridae[31]. Three phylogenetically distinct genotypes of HDV that have different geographic distributions and different disease severities have been reported[35,36,37].

## The Virus Particle (HDV Virion)

The virion particle is approximately 36 nm in size[31]. It is composed of an outer envelope that encloses a nucleocapsid (Fig. 4). The outer envelope is contributed by its helper HBV and is composed of hepatitis B surface antigen (HBSAg)[31].

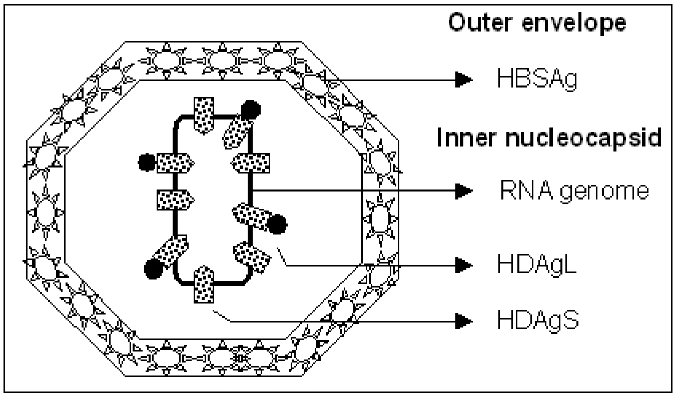

**FIGURE 4**. Schematic representation of the HDV virion. It has an outer envelope, comprised of HBSAg, and an inner nucleocapsid. The nucleocapsid contains the RNA genome and two forms of HDAg: HDAgL (the long form) and HDAgS (the short form).

The nucleocapsid of HDV is composed of a negative-stranded RNA genome that is tightly bound to the viral protein, hepatitis delta antigen (HDAg)[31].

## The HDV Genome and Viral Gene Products

The RNA genome of HDV (HDV RNA) is a minus-sense, single-stranded, covalently closed circular molecule that replicates by the double-rolling circle mechanism, using host enzymes[31,38]. It has only one functional open reading frame (ORF) that is present on the antigenomic RNA and codes for the viral protein, HDAg[39,40].

Being approximately 1.7 kb in size, the HDV RNA is the smallest genome present in a known human pathogen[39,40]. An important feature of this RNA is that, even though it is a single-stranded circular molecule, about 70% of its sequences are base paired. Therefore it appears like a rod-shaped dsRNA[39,40]. This dsRNA-mimicking feature of HDV RNA makes it an ideal substrate for the ADAR enzymes[39,40].

## ADAR Activity on HDV RNA

The deamination activity of ADAR1[41] is required for completing the infectious lifecycle of HDV; ADAR-effected deamination (RNA editing) at a specific site (1012A) on the antigenomic HDV RNA facilitates the virus to diversify its codon output and to produce a variant form of HDAg that is essential for virion packaging (Fig. 5).

The diversification of HDV's codon output occurs because site 1012A is the second base present on the amber stop codon (UAG) of the HDAg ORF. Therefore, when 1012A is edited, the amber stop codon (UAG) is transformed to a tryptophan (W) codon (UGG). This transformation extends the HDAg ORF by 19 amino acids at the C-terminus, and results in the production of a variant (longer) HDAg protein[31]. In agreement with their physical characteristics, this longer version, which is the product of RNA editing at 1012A, is referred to as HDAgL ("L" for Long form) and the shorter version which is coded from the unedited ORF is called HDAgS ("S" for Short form). Also, due to the specific codon transformation from amber stop to W, the editing at 1012A is referred to as amber/W editing, and 1012A is called amber/W site.

The S and L forms of HDAg, although different only in their C-terminal amino acids, serve very different and distinctive roles in HDV lifecycle. HDAgS, a 24-kD protein having 195 amino acids, is indispensable for HDV genome replication[42], whereas the 27-kD HDAgL protein that has 214 amino acids is required for HDV virion packaging, but has no supportive role in viral genome replication[42,43]. In fact, HDAgL inhibits HDV RNA replication and can act as a dominant negative inhibitor of the replication-supportive effect of HDAgS[42,43]. Therefore, during the HDV lifecycle, the virus must be able to circumvent the replication-inhibitory effect of HDAgL so that genome replication progresses until sufficient amounts of genomic RNA have been synthesized for packaging. However, at the time of virion assembly, adequate amounts of HDAgL must be available for packaging.

On par with these logistics, from our understanding about HDV biology we know that even though ADAR activity is essential to complete the virus lifecycle, the virus has to enforce at least three types of regulation over ADAR activity on its RNA:

- Spatial regulation and specificity: deamination on HDV RNA has to be targeted to 1012A specifically. Nonspecific random editing can deter virus viability by compromising the RNA's replication ability and also by creating dominant negative inhibitors of replication[43].

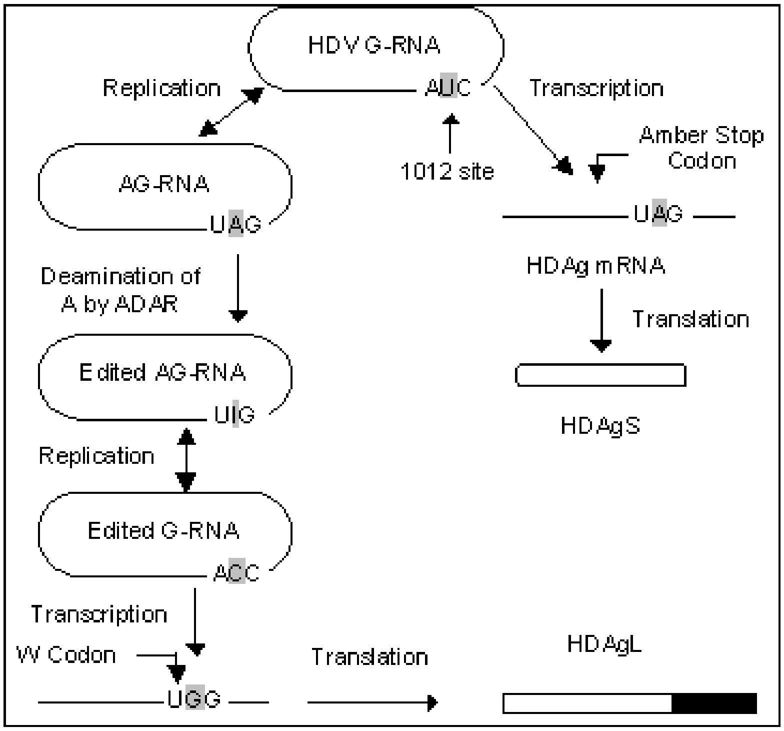

**FIGURE 5**. ADAR activity on HDV RNA. (G-RNA: genomic RNA. AG-RNA: antigenomic RNA. W: tryptophan). During HDV replication, by the double-rolling circle mechanism, the G-RNA replicates to form AG-RNA and vice versa. Simultaneously, mRNA for HDAg is transcribed off the G-RNA. This mRNA is translated to form the protein referred to as HDAgS (short form). Later in the replication cycle, ADAR enzymes act on part of the AG-RNA molecules, causing deamination of A on the UAG stop codon I. Subsequent rounds of replication forms edited G-RNAs, which are then transcribed to mRNAs that have their UAG stop codons converted to UGG W codons, and hence having longer reading frames. Proteins translated from these edited mRNAs are longer compared to HDAgS, and are called HDAgL (long form).

- Temporal regulation: significant levels of editing must not occur until replication has produced sufficient amounts of HDV RNA genome that can be packaged into virions. This is because accumulation of the editing product (HDAgL) could suppress RNA replication and genome synthesis[44]. In agreement with this rationale, maximum editing is seen only at the later stages during the HDV lifecycle: cell culture studies show that peak editing is seen about 10–12 days after the initiation of replication[45,46].
- Quantitative regulation: the level of editing must not exceed a point where overproduction of HDAgL might compromise RNA replication and therefore virus viability[43]. Supportive of this rationale, during the HDV lifecycle, only 15–30% of the HDV RNA population is amber/W-edited (i.e., the maximum level of amber/W editing is 15–30%).

Even though we know the above facts about HDV biology, it is still not clear how the virus is able to exert such tight control over the host ADAR enzymes.

There are several unanswered questions: What mechanism does this tiny satellite virus, which is totally dependent on host enzymes for its replication, use to enforce such a tight regulatory paradigm on the host ADAR enzymes? Could there be host proteins that interact with HDV to facilitate such regulation? This possibility cannot be ruled out, because Don Ganem's group has identified a mammalian host protein, DIPA (delta interacting protein A), that can interact with HDAg-S, and can also modulate HDV replication[47]. There could well be other delta-interacting proteins that may act as HDV-helpers, to facilitate regulated interaction of HDV and HDV RNA with host enzymes such as ADARs. Further studies are warranted to answer these questions.

## PERSPECTIVE

It is over 2 decades since HDV was first reported and discovered by Rizzetto's group[48], but several features of this viriod-like human virus are yet to be unraveled. For example, HDV does not encode viral polymerase or reverse transcriptase. It is believed to use the host polymerase (Pol II) to replicate its RNA genome. However, we have no clue as to how the virus is able to convince the host polymerase, which normally acts on DNA substrates, to transcribe from its RNA template.

Another fascinating aspect about this virus is its interaction with ADAR enzymes. We know that regulated ADAR1[41] activity, directed specifically to the amber/W site, is essential for HDV lifecycle. However, it is still a mystery as to how the virus achieves amber/W site specificity, without subjecting the other 200 adenosines in the HDAg-coding region to ADAR-induced deamination. Also, how are the optimum levels of deamination at the amber/W site regulated? This is all the more intriguing considering the more recent reports on the interferon-inducible nature of ADARs and the proposed role of ADARs in cellular inflammatory and antiviral pathways[49,50,51].

It has been hypothesized that the dynamic secondary structure of the HDV RNA is a major determinant of the virus's ability to interact with host factors. Studies have shown that specific secondary structures on the HDV RNA, and the dexterity of the RNA to switch between conformations, are critical for its controlled and varied interaction with HDAgS and with host proteins[52,53] and could therefore be critical for the regulatory events associated with HDV lifecycle[46,54]. However, the mechanistic and functional aspects underlying these phenomena are yet to be understood and studies aimed at elucidating these details are warranted for several reasons:

- A better understanding of the regulatory mechanisms associated with HDV lifecycle and the host and viral factors involved in these processes will be useful for developing antiviral strategies against delta hepatitis and against other RNA virus infections.
- Gaining a better perspective about the interaction of HDV with ADAR and other host proteins would also enhance our understanding of the biology and pathogenesis of its helper hepatitis B virus and thereby facilitate the development of improved therapies for hepatitis B infection.
- Since HDV genome has great resemblance to the genome of plant viroids that are responsible for a variety of plant viral epidemics[47], understanding HDV biology would have a wider global significance for counteracting plant pathogens too.
